# Identifying *Chloris* Species from Cuban Citrus Orchards and Determining Their Glyphosate-Resistance Status

**DOI:** 10.3389/fpls.2017.01977

**Published:** 2017-11-15

**Authors:** Enzo R. Bracamonte, Pablo T. Fernández-Moreno, Fernando Bastida, María D. Osuna, Ricardo Alcántara-de la Cruz, Hugo E. Cruz-Hipolito, Rafael De Prado

**Affiliations:** ^1^Faculty of Agricultural Sciences, National University of Cordoba (UNC), Cordoba, Argentina; ^2^Department of Agricultural Chemistry and Edaphology, University of Cordoba, Cordoba, Spain; ^3^Department of Agroforestry Sciences, University of Huelva, Huelva, Spain; ^4^Agrarian Research Center “Finca La Orden Valdesequera”, Badajoz, Spain; ^5^Departamento de Entomologia/BIOAGRO, Universidade Federal de Viçosa, Viçosa, Brazil; ^6^Bayer CropScience, Mexico City, Mexico

**Keywords:** 5-enolpyruvyl shikimate-3-phosphate synthase, glyphosate translocation, herbicide resistance mechanisms, Pro-106 mutation, tall windmill grass

## Abstract

The *Chloris* genus is a C_4_ photosynthetic species mainly distributed in tropical and subtropical regions. Populations of three *Chloris* species occurring in citrus orchards from central Cuba, under long history glyphosate-based weed management, were studied for glyphosate-resistant status by characterizing their herbicide resistance/tolerance mechanisms. Morphological and molecular analyses allowed these species to be identified as *C. ciliata* Sw., *Chloris elata* Desv., and *Chloris barbata* Sw. Based on the glyphosate rate that causes 50% mortality of the treated plants, glyphosate resistance (R) was confirmed only in *C. elata*, The R population was 6.1-fold more resistant compared to the susceptible (S) population. In addition, R plants of *C. elata* accumulated 4.6-fold less shikimate after glyphosate application than S plants. Meanwhile, populations of *C. barbata* and *C. ciliata* with or without glyphosate application histories showed similar LD_50_ values and shikimic acid accumulation rates, demonstrating that resistance to glyphosate have not evolved in these species. Plants of R and S populations of *C. elata* differed in ^14^C-glyphosate absorption and translocation. The R population exhibited 27.3-fold greater 5-enolpyruvyl shikimate-3-phosphate synthase (EPSPS) activity than the S population due to a target site mutation corresponding to a Pro-106-Ser substitution found in the EPSPS gene. These reports show the innate tolerance to glyphosate of *C. barbata* and *C. ciliata*, and confirm the resistance of *C. elata* to this herbicide, showing that both non-target site and target-site mechanisms are involved in its resistance to glyphosate. This is the first case of herbicide resistance in Cuba.

## Introduction

The use of herbicides is the most common weed control method (Délye, 2013; Fernández-Moreno et al., 2017b). However, herbicide resistance has caused this method to be quickly undermined. This scenario is the result of evolutionary adaptations in a target weed to herbicide applications (Powles and Yu, 2010; Beckie and Harker, 2017). Glyphosate [(N-phosphonomethyl)-glycine] is one of the most widely used herbicides, although it is also an herbicide with many cases of resistance (37 glyphosate-resistant species; Shaner et al., 2012; Bracamonte et al., 2016; Heap, 2017). This herbicide is systemic, non-selective and is used post-emergence, and it inhibits the 5-enolpyruvylshikimate-3-phosphate synthase (EPSPS) gene (EC 2.5.1.19), triggering the catalysis of shikimate-3-phosphate and phosphoenolpyruvate (PEP) to form 5-enolpyruvyl-3-phosphate, an important step in the biosynthesis of aromatic amino acids in plants (Schönbrunn et al., 2001).

The mechanisms conferring glyphosate resistance are grouped into two major groups (Sammons and Gaines, 2014). Target-site resistance (TSR) can involve EPSPS gene mutations or EPSPS gene amplification. TSR was revealed to be caused by point mutations in the EPSPS gene with substitutions at Thr-102 and Pro-106 (Yu et al., 2015; Alcántara-de la Cruz et al., 2016b). Pro-106 substitutions have been found in several weeds (González-Torralva et al., 2012; Alarcón-Reverte et al., 2015; Fernandez et al., 2015), conferring low resistance levels to glyphosate on the order of 2- to 5-fold, while a double mutation (Thr-102 and Pro-106) increases resistance levels. Gene amplification is an adaptation also confers resistance to glyphosate (Gaines et al., 2013). The additional EPSPS produced from the amplified gene copies enables plants to survive higher glyphosate doses (Gaines et al., 2013; Chen et al., 2015; Yu et al., 2015). Non-target-site resistance (NTSR) mechanism results from reduced absorption and/or translocation, increased vacuolar sequestration, and metabolism to non-toxic compounds, causing less glyphosate transport to the EPSPS via the xylem and phloem (Délye, 2013). NTSR has being described as the most common mechanism of resistance to glyphosate and can confer unpredictable resistance (Powles and Yu, 2010). Similar to TSR, NTSR has been found to be a mechanism involved in resistance in many weeds (de Carvalho et al., 2012; Ge et al., 2012; Rojano-Delgado et al., 2012; Vila-Aiub et al., 2012).

The genus *Chloris* Sw. (Poaceae: Chloridoidae) is a C_4_ photosynthetic species distributed in tropical and subtropical regions (Molina and Agrasar, 2004). It has also been found in semi-arid areas inhabiting semi-natural grasslands and rural habitats such as roads and barren places (Cerros-Tlatilpa et al., 2015). The genus comprises 50–60 species in both hemispheres (Molina and Agrasar, 2004; Barkworth, 2007). Species of this genus have great economic and ecological importance worldwide because they are a source of forage, resist drought, increase soil fertility, require low inversion, and can be used as plant cover to protect soil from rain-driven erosion (Michael et al., 2012). However, some of them could be considered invasive weed species (Cerros-Tlatilpa et al., 2015).

Studies of herbicide resistant weeds in Cuba are scarce because of lack of knowledge of the issue. This situation is similar to the Dominican Republic, in which studies have already begun to be carried out (Bracamonte et al., 2016). Unfortunately, growers have already started to have many weeds in their citrus groves that are not controlled at the recommended field dose of glyphosate (720 g ae ha^−1^). Given that weed control strategies in larger commercial fields are absolutely the focus of glyphosate applications at the post-emergence growth stage, scientific confirmation is necessary. In this way, this study could define the species that are evolving toward to glyphosate resistance or even tolerant species to this herbicide.

An accurate assessment of taxonomic identity is a prerequisite to addressing population and individual plant-based functional studies. This is particularly true in the case of highly diverse genera, for which taxonomic and nomenclatural complexities generally arise, as is the case in *Chloris* (Molina and Agrasar, 2004).

This work aimed to characterize suspicious glyphosate-resistant populations of three different *Chloris* species in Cuba. Studies were conducted to (1) establish their taxonomical identity based on morphological and molecular analyses, (2) evaluate their resistance/tolerance levels, and (3) determine the mechanisms involved.

## Material and methods

### Plant material and experimental conditions

In 2014, our research group (Dr. Rafael De Prado) together with the Weed Science group of the Ministry of Agriculture of Cuba (Dr. Jorge Cueto), prospected for *Chloris* species in citrus orchards in central Cuba. These fields had been repeatedly treated with glyphosate (5 L ha^−1^, 36% w/v) continuously for over 10 years, and sometimes received more than one application per year (Cueto, personal communication).

Mature seeds of three suspicious glyphosate-resistant populations of *Clhoris* species (treated = T) were harvested separately in in citrus orchards from Arimao and Ceiba, in Cienfuegos Province, from at least 20 plants that had been survived to the last glyphosate treatment. Seeds from a population of each species from nearby locations with no known records of exposure to glyphosate were also collected (non-treated = NT).

The seeds were germinated in containers using a substrate of sand/peat (1:2 v/v), covered with parafilm, and placed in a growth chamber at temperatures of 28/18°C (day/night), with a 16 h photoperiod (850 μmol m^−2^ s^−1^) and 80% humidity. Subsequently, the seedlings from each population of the different *Chloris* species were transplanted individually into pots (1 plant per pot) containing the same substrate and placed in a growth chamber under the conditions described above. Furthermore, 20–30 plants from each population were placed in a greenhouse until flowering and fruiting.

### Morphometric study and taxonomic identity

Different taxonomically relevant morphological traits of inflorescences and caryopses were measured in greenhouse-grown plants of each species of the *Chloris* populations. The examined traits of inflorescences were the number of racemes, raceme length and spikelet density (number of spikelets per cm of raceme). For the spikelets, we examined the length and width of the lower and upper glumes, the number of sterile florets, the length of hairs surrounding the callus, the length and width of the lemma of the fertile floret (fertile lemma), the presence and length of hairs on or adjacent to the keel and on the margins of fertile lemma, the length of the palea of the fertile floret, the lemma length and width of the basal sterile floret, the lemma length of any additional sterile floret, and the presence and length of awns on lemmas. The characteristics of the caryopses were length, width, thickness, shape, and length of the embryo mark. The shape of caryopses was quantified as the variance in their three dimensions, each relative to length (Thompson et al., 1993). This dimensionless shape index varies between 0 for a perfect sphere and 0.22 for a disk- or needle-shaped item. Based on the above morphological characters, the three pairs of study populations were identified to the species level according to Molina and Agrasar (2004), and the nomenclature followed IPNI (2017).

### Molecular characterization of the *Chloris* species by AFLP primer analysis

Twenty-four accessions from the *Chloris* spp. were employed as the study material. Genomic DNA was extracted from fresh young leaves of eight individual plants per species (four T and four NT), using the Speedtools Plant DNA Extraction kit (Biotools). The DNA concentration was measured using a NanoDrop ND 1000 spectophotometer. DNA was diluted to a final concentration of 10 ng/μL.

Twelve AFLP primer pairs were used [E36-M48 (E-ACC M-CAC); E36-M60 (E-ACC M-CTC); E37-M49 (E-ACG M-CAG); E38-M50 (E-ACT M-CAT); E40-M61 (E-AGC M-CTG); E35-M49 (E-ACA M-CAG); E36-M49 (E-ACC M-CAG); E35-M61 (E-ACA M-CTG); E40-M62 (E-AGC M-CTT); E32-M60 (E-AAC M-CTC); E33-M50 (E-AAG M-CAT)]. The reaction mix contained 10 ng template DNA, 2.5 U Taq DNA polymerase, 40 pmol primer, 200 μM dNTPs, 2.5 mM MgCl_2_, and 10 mM Tris-HCl all in a volume of 20 μl. The optimized thermal cycling conditions were 2 min at 94°C, followed by 40 cycles of 94°C for 25 s, 56°C for 25 s, 72°C for 25 s and a final extension at 72°C for 7 min. AFLP fragments were resolved in 25-cm gels (0.25 mm spacer thickness). Electrophoresis and detection were performed on a two-dye, model 4300 LICOR automated DNA Sequencer. Digital AFLP gel images were scored to obtain binary (band presence/absence) data using the SAGA GENERATION 2 software program.

Data clustering was conducted for AFLPs with the NTSYS-pc-2.2 software (Rohlf, 2000) using Jaccard's coefficients to define unweighted pair-group (UPGMA) dendograms. A principal coordinate analysis (PCA) was also performed with the NTSYS-pc program.

### Dose-response assays

Plants of each *Chloris* population were sprayed at the 3–4 leaf growth stage. Glyphosate applications were applied with a laboratory chamber (SBS-060 De Vries Manufactering, Hollandale, MN) equipped with 8002 flat fan nozzle delivering 200 L ha^−1^ at 250 KPa at the height of 50 cm. The following glyphosate (Roundup®, 360 g ae L^−1^ as isopropylamine salt) rates were used: 0, 62.5, 125, 250, 500, 1,000, 2,000, 3,000, and 4,000 g ae ha^−1^. The experiment was design using nine replications per rate and was repeated twice. Plants were cut down at the soil surface 21 days after application (DAT).

### Shikimic accumulation assay

Fifty mg of fresh tissue (4 mm leaf disks) were harvested from the youngest fully expanded leaf at the 3–4 leaf growth stage from 15 plants per population. Shikimic acid accumulation was determined according to Hanson et al. (2009). The glyphosate concentrations used were: 0, 500, and 1,000 μM. Sample absorbance was measured in a Beckman DU-640 spectrophotometer at 380 nm. The test was performed in triplicate on five treated and non-treated plants of each biotype in a completely random design. Results were expressed in mg of shikimic acid g^−1^ fresh tissue.

### Absorption and translocation

This study was carried out in the two *C. elata* populations.^14^C-glyphosate (American Radiolabeled Chemicals, Inc., USA) was added to the commercial herbicide to prepare a solution with a specific activity of 0.834 kBq μL^−1^. The final glyphosate concentration corresponded to 360 g ae ha^−1^ in 200 L ha^−1^. Plants were harvested at 24, 48, 48, 72, and 96 h after ^14^C-glyphosate treatment (0.834 kBq/plant). Five plants per populations at each time evaluated in a completely random design were handled according to Fernández-Moreno et al. (2017b). Radioactivity was analyzed by liquid scintillation spectrometry (LSS) in a Beckman LS 6500 scintillation counter (Beckman Coulter Inc. Fullerton, USA) during 10 min per sample. Percentage of ^14^C-glyphosate absorbed was expressed as [kBq in combusted tissue/(kBq in combusted tissue + kBq in leaf washes)] × 100.

Translocation of ^14^C-glyphosate in plants of the two *C. elata* populations was visualized using a phosphor imager (Cyclone, Perkin-Elmer, Waltham, MA, USA).

### Glyphosate metabolism

Six plants by each *C. elata* population at 3–4 leaf growth stage, were treated with 300 g ae ha^−1^ of glyphosate (as described in the dose-response assays) in a completely randomized design. Untreated plants were used as controls. Leaf tissues were washed with distilled water at 96 HAT, flash-frozen in liquid nitrogen, and stored at −40°C until use. Following the methodology described by Rojano-Delgado et al. (2010), glyphosate and its metabolites [aminomethyl phosphonate (AMPA), glyoxylate, sarcosine, and formaldehyde] were determined by reversed polarity capillary electrophoresis using a 3D Capillary Electrophoresis Agilent G1600A instrument equipped with a diode array detector (DAD, wavelength range 190–600 nm). Standard compounds used (glyphosate, AMPA, sarcosine, formaldehyde, and glyoxylate), were provided by Sigma-Aldrich, Spain. Glyoxylate naturally produced (untreated plants) was subtracted from the average of glyoxylate produced from glyphosate metabolism (treated plants) for each population.

### EPSPS enzyme activity assays

Leaf tissue of the *C. elata* populations (three samples of 5 g each) were ground to fine powder in liquid nitrogen a chilled mortar. The enzyme activity was extracted according to the protocol described by Sammons et al. (2007). The basal EPSPS activity in the extract was measured using a Modified Lowry Kit for Protein Determination (Sigma-Aldrich, Madrid, Spain) in accordance with the manufacturer's instructions. The specific EPSPS activity was determined using the EnzCheck Phosphate Assay Kit (Invitrogen, Carlsbad, CA) following the manufacturer's instructions, to determine the inorganic phosphate release. The glyphosate concentrations used were: 0, 0.1, 1, 10, 100, and 1,000 μM. The EPSPS activity was measured during 10 min at 360 nm in a spectrophotometer (Beckman DU-640) to determine the amount of phosphate (μmol) released μg of total soluble protein (TSP)^−1^ min^−1^ and expressed as a percentage with respect to the control (without glyphosate). The experiment was repeated three times for each samples.

### EPSPS gene sequence

Young tissue (100–200 mg) was collected from 10 plants of each *C. elata* population and stored at −80°C for RNA extraction. Total RNA was isolated using the TRIzol reagent (Invitrogen, Carlsbad, CA, USA) according to the manufacturer's instructions. RNA was then treated with TURBO DNase (RNase-Free; Ambion, Warrington, UK) to eliminate any DNA contamination. cDNA synthesis was carried out using 2 μg of total RNA and M-MLV (Moloney Murine Leukemia Virus) Reverse Transcriptase (Invitrogen, Carlsbad, CA, USA) in combination with oligo (dT)_12−18_ and random nonamers (Amersham Biosciences, Amersham, UK) according to the manufacturer's instructions. To amplify the EPSPS gene, primers previously designed by Perez-Jones et al. (2007) (forward: 5′ AGCTGTAGTCGTTGGCTGTG 3′; reverse: 5′ GCCAAGAAATAGCTCGCACT 3′), and de Carvalho et al. (2012) (forward: 5′ TAGTACAGCCAAAAGGGCAGTC-3′; reverse: 5′ GCCGTTGCTGGAGGAAATTC 3′) were used. These primers expand a 120-bp fragment of the EPSPS gene that contains the mutation site described as conferring resistance to glyphosate. The PCR reactions were carried out using cDNA from 50 ng of total RNA, 1.5 mM MgCl_2_, 0.2 mM dNTP, 0.2 μM of each primer, 1 × buffer, and 0.625 units of a 100:1 enzyme mixture of non-proofreading (*Thermus thermophilus*) and proofreading (*Pyrococcus furiosus*) enzymes (BIOTOOLS, Madrid, Spain) in a final volume of 25 μL. All PCR reactions were completed in duplicate, and the cycling conditions were as follows: 94°C for 3 min, 35 cycles of 94°C for 30 s, 55°C for 30 s and 72°C for 1 min, with a final extension cycle of 72°C for 10 min. An aliquot of the PCR product was loaded onto a 1% agarose gel to confirm the correct band amplification. The remainder of the PCR product was then purified using ExoSAP-IT® for PCR Product Clean-Up (USB, Ohio, USA) as indicated by the manufacturers. Five purified PCR products per population were sequenced (STAB VIDA, Caparica, Portugal).

### Statistical analysis

Dose-response and EPSPS enzyme activity data were subjected to non-linear regression analysis to determine the amount of glyphosate needed to reduce the fresh weight (GR_50_), increase the mortality (LD_50_), and inhibit the EPSPS activity (I_50_) by 50% in each *Chloris* population using the three-parameter log-logistic function: *y* = ([(*d*) / 1+(*x*/*g*)^*b*^]) where *y* is, depending on the analysis, the above ground fresh weight, survival, or EPSPS-activity expressed as the percentage of the non-treated control, *d* is the parameter corresponding to the upper asymptote, *b* is the slope, *g* is the GR_50_, LD_50_, or I_50_, and *x* (independent variable) is the glyphosate rate. Regression analyses were conducted using the *drc* package in the R program version 3.2.5 (Ritz et al., 2015). Resistance indexes (RI = R/S) were computed as R-to-S GR_50_, LD_50_, or I_50_ ratios.

An analysis of variance (ANOVA) was conducted to test for differences between populations in the different assays. When needed, differences between means were separated using the Tukey HSD test at *P* < 0.05. Model assumptions of the normal distribution of errors and homogeneous variance were graphically inspected. The ANOVAs were conducted using the Statistix (version 9.0) (Analytical Software, USA) software.

## Results

### Morphometric study and taxonomic identity

Based on the examined morphological traits, the populations studied were identified as *C. ciliata* Sw., *C. elata* Desv., and *Chloris barbata* Sw. Only the last species listed is an annual species, and it can be easily separated from the other two species by its long-awned lemmas, the glabrous keel of the fertile lemma and the presence of hairs flanking the keel. In addition, caryopses of *C. barbata* were clearly more elongated in shape than those of the remaining species, as indicated by the higher values of the seed shape index. Distinctive traits of *C. ciliata* include a low number of racemes in the inflorescences, more than two sterile florets per spikelet, long keel hairs and relatively short awns. Compared to the other two species, racemes of *C. elata* plants were consistently longer and their fertile lemmas were shorter in length, showing much longer marginal hairs. In addition, the embryo mark in caryopses was shorter in this species (Table 1).

**Table 1:** Comparison of morphological traits from inflorescences and caryopses for the three pairs of studied *Chloris* populations, and their taxonomic identity at the species level.

**Species**		***C. ciliata* T**	***C. ciliata* NT**	***C. elata* T**	***C. elata* NT**	***C. barbata* T**	***C. barbata* NT**
Number of racemes composing inflorescences		5 (5–7)	5 (4–5)	17–28	11–27	13 (11-15)	12–18
Racemes length		54.6 ± 6.8	76.0 ± 12.0	117.0 ± 20.9	96.0 ± 16.9	71.2 ± 8.5	73.0 ± 9.2
Number of spikelet per cm raceme		14 (14–14)	14 (12–14)	11 (9–11)	14 (12–15)	12 (12–13)	13 (12–13)
Number of sterile florets in spikelets		3 (3–4)	4 (3–4)	2 (2–2)	2 (2–2)	2 (2–2)	2 (2–2)
Length of hairs surrounding spikelet callus		0.66 ± 0.21	0.79 ± 0.12	0.18 ± 0.03	0.40 ± 0.06	0.95 ± 0.08	0.97 ± 0.12
Fertile floret, lemma	Length	2.68 ± 0.19	2.65 ± 0.18	1.98 ± 0.11	1.87 ± 0.10	2.63 ± 0.12	2.54 ± 0.10
	Max width	2.14 ± 0.19	2.18 ± 0.20	1.36 ± 0.09	1.56 ± 0.10	1.18 ± 0.17	1.13 ± 0.14
	Hairy (H)/glabrous (G) keel	H	H	H	H	G	G
	Length of keel hairs	1.20 ± 0.15	1.30 ± 0.15	0.67 ± 0.05	0.70 ± 0.06	–	–
	Length of hairs flanking keel	–	–	–	–	0.57 ± 0.08	0.58 ± 0.14
	Length of marginal hairs	1.13 ± 0.15	1.42 ± 0.26	2.22 ± 0.16	2.09 ± 0.10	1.40 ± 0.13	1.40 ± 0.06
	Awn length	1.63 ± 0.26	1.71 ± 0.35	3.14 ± 0.36	2.53 ± 0.27	6.97 ± 0.60	5.53 ± 1.08
Fertile floret, palea	Length	2.44 ± 0.14	2.55 ± 0.20	1.82 ± 0.09	1.58 ± 0.09	2.45 ± 0.14	2.35 ± 0.23
Basal (first) sterile floret, lemma	Length	1.66 ± 0.14	1.73 ± 0.18	1.09 ± 0.08	1.05 ± 0.05	1.38 ± 0.12	1.37 ± 0.05
	Max width	2.15 ± 0.21	2.23 ± 0.23	0.92 ± 0.10	0.97 ± 0.08	1.27 ± 0.28	1.33 ± 0.08
	Awn length	1.58 ± 0.26	1.72 ± 0.23	2.83 ± 0.15	2.22 ± 0.29	6.37 ± 1.23	5.33 ± 1.89
Second sterile floret, lemma	Length	1.14 ± 0.17	1.08 ± 0.15	0.50 ± 0.05	0.53 ± 0.04	1.29 ± 0.09	1.33 ± 0.08
	Awn length	A	A	A	A	5.22 ± 0.81	3.82 ± 0.88
Glumes	Upper, length	2.57 ± 0.17	2.78 ± 0.07	3.10 ± 0.06	3.25 ± 0.20	2.82 ± 0.08	2.78 ± 0.12
	Upper, width	1.00 ± 0.19	0.92 ± 0.13	0.53 ± 0.05	0.45 ± 0.14	0.60 ± 0.06	0.55 ± 0.05
	Lower, length	1.75 ± 0.15	1.73 ± 0.08	2.07 ± 0.13	2.03 ± 0.14	1.78 ± 0.08	1.80 ± 0.06
	Lower, width	0.83 ± 0.10	0.78 ± 0.04	0.73 ± 0.08	0.80 ± 0.06	0.58 ± 0.08	0.45 ± 0.05
Caryopsis	Length	1.43 ± 0.08	1.35 ± 0.12	1.12 ± 0.04	1.03 ± 0.05	1.45 ± 0.05	1.40 ± 0.00
	Width	0.70 ± 0.04	0.58 ± 0.09	0.57 ± 0.04	0.60 ± 0.00	0.45 ± 0.03	0.43 ± 0.03
	Thickness	0.58 ± 0.04	0.53 ± 0.07	0.45 ± 0.03	0.47 ± 0.03	0.33 ± 0.03	0.31 ± 0.02
	Length of the embryo mark	0.83 ± 0.08	0.77 ± 0.09	0.59 ± 0.07	0.62 ± 0.13	0.93 ± 0.03	0.90 ± 0.05
	Seed shape index^a^	0.069 ± 0.008	0.078 ± 0.001	0.068 ± 0.005	0.055 ± 0.008	0.121 ± 0.004	0.122 ± 0.005

a *Variance of the three dimensions, each relative to length*.

*Means and standard deviations are given for quantitative traits, and modal (if any), minimum and maximum values for qualitative traits. Sample size n = 6. Linear dimensions are given in mm. A, absent*.

### Molecular characterization of the genus *Chloris*

**Figure 1:**
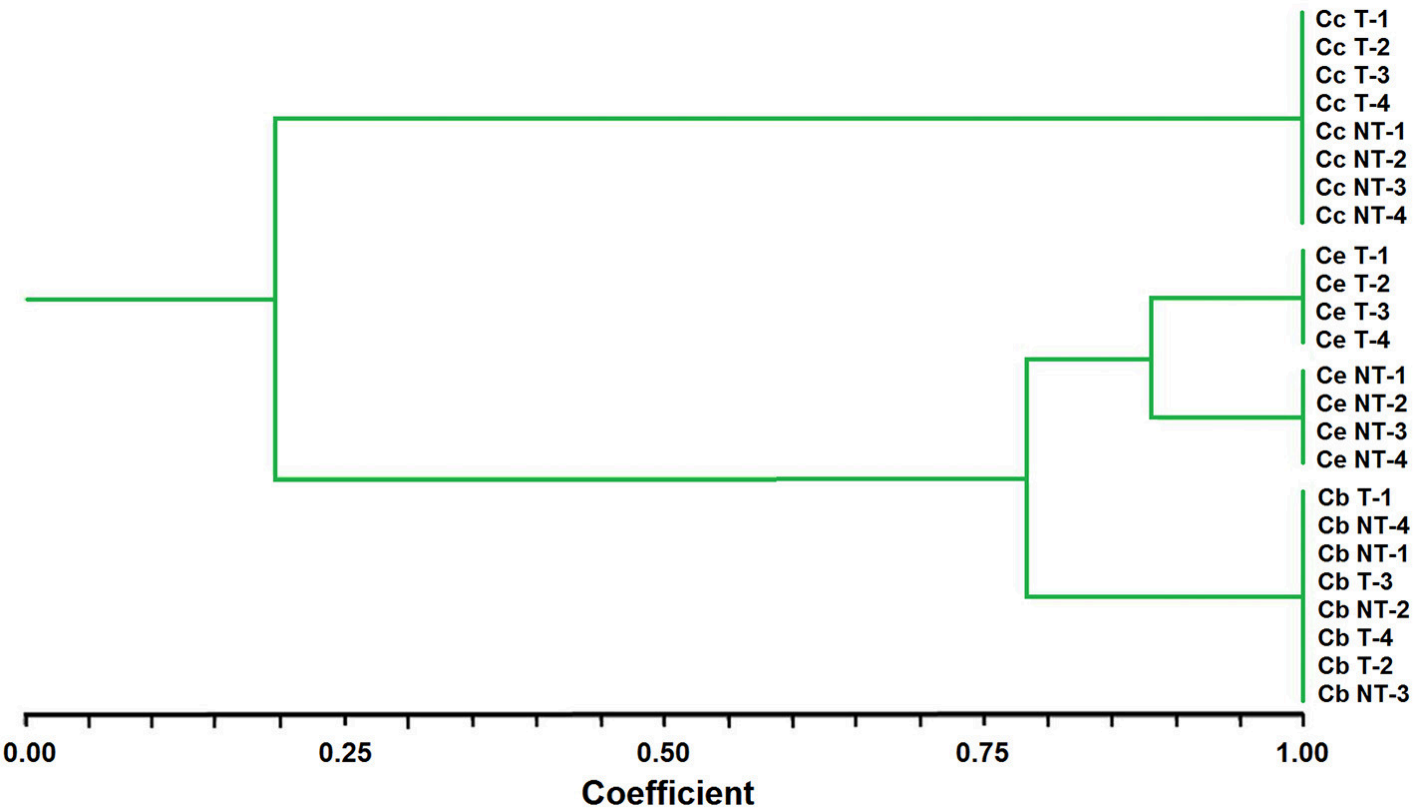
Genetic similarities among *Chloris* species after UPGMA analysis performed with AFLP marker data. Ce, *Chloris elata*; Cb, C. *barbata*; and Cc, *C. Ciliata;* T, plants with glyphosate history applications; NT, non-treated plants.

A cluster analysis using UPGMA methods classified the *Chloris* populations into two major groups (I and II), thus providing complementary information to the morphological analysis. Group I contained all samples of *C. ciliata* whereas Group II consisted of two subgroups, II-1 including *C. elata*, and II-2 including *C. barbata*. It is noting that the cluster analysis did not separate T and NT populations of *C. ciliata* or *C. barbata*, while the T and NT populations of *C. elata* were clearly separated (Figure 1).

### Dose-response assays

**Figure 2:**
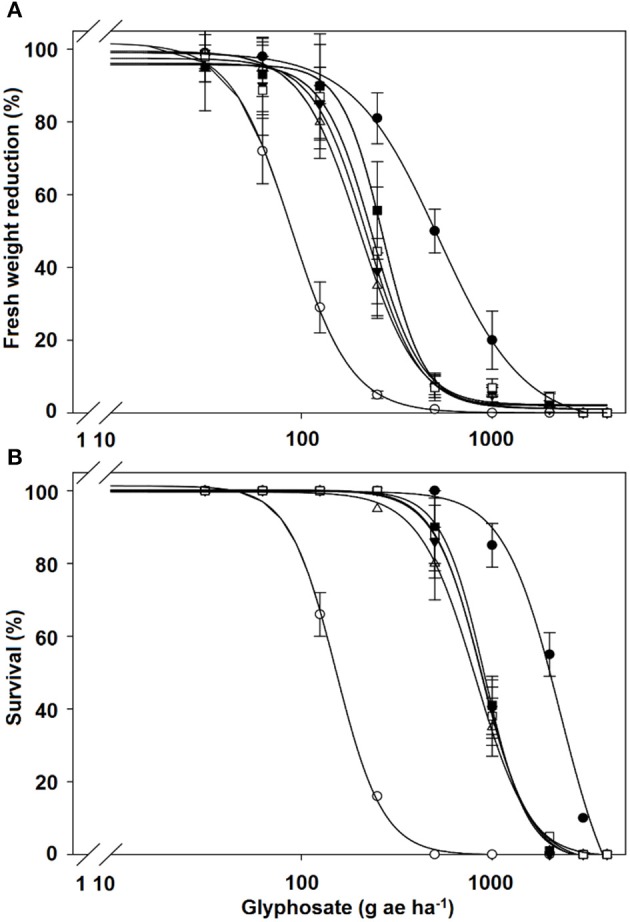
Glyphosate **(A)** dose-response on fresh weight reduction and **(B)** % survival expressed as percentage respect to untreated control of the treated (T) (•) and non-treated (NT) (○) populations of *Chloris elata*; T (▾) and NT (Δ) populations of *C. barbata*; and T (■) and NT (□) populations of *C. ciliata*. Vertical bars are ± standard errors (*n* = 9).

The fresh weight reduction by 50% (GR_50_) for the NT and T populations of *C. elata* was achieved at 88.3 and 542.1 g ae ha^−1^, respectively, i.e., T populations were 6.1-fold more resistance to glyphosate than the NT population. The LD_50_ values of the T populations exhibited 15-fold resistance than the NT population of *C. elata*. In contrast, GR_50_ and LD_50_ values in both *C. barbata* and *C. ciliata* species were not different between T and NT populations (Table 2, Figure 2).

**Table 2:** Glyphosate rates required for 50 % reduction fresh weight (GR_50_) and survival (LD_50_) expressed as percentage of the mean untreated control of *Chloris* species.

**Species**	**Status^a^**	**GR_50_ (g ae ha^−1^)**	**RI^b^**	***P***	**LD_50_ (g ae ha^−1^)**	**RI^b^**	***P***
*C. elata*	T	542.1 ± 31.3	6.1	0.001	2277.7 ± 245.1	15.0	0.001
	NT	88.3 ± 4.8			151.6 ± 24.8		
*C. barbata*	T	217.2 ± 19.6	1.1	0.241	889.4 ± 71.5	1.1	0.382
	NT	198.9 ± 21.9			820.2 ± 79.0		
*C. ciliata*	T	263.1 ± 19.2	1.1	0.159	912.7 ± 68.6	1.1	0.297
	NT	231.3 ± 37.4			875.2 ± 59.3		

*±Standard error (n = 10)*.

a *Status: T, populations with glyphosate history applications; and NT, non-treated populations with glyphosate*.

b *RI, Resistance index (R/S) calculated using the corresponding ED_50_, or LD_50_ values of the resistant populations respect to the susceptible one*.

### Shikimic acid accumulation

**Figure 3:**
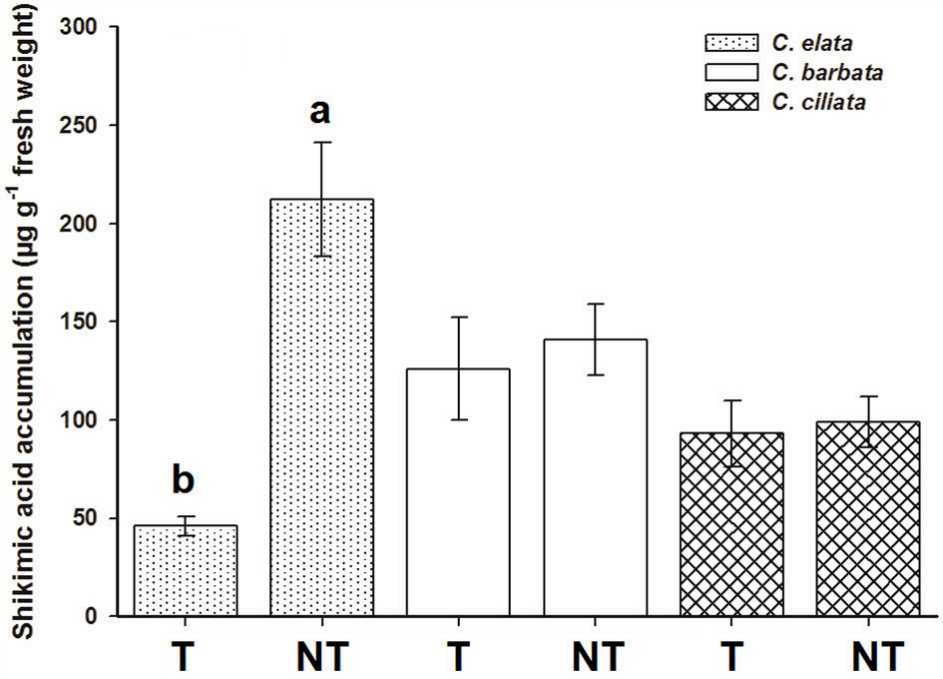
Shikimic acid accumulation in *Chloris* spp. plants at 1,000 μM glyphosate concentration. T = plants with glyphosate history applications; and NT = non-treated plants. Vertical bars are ± standard errors (*n* = 5). Different letters shown differences at 95% by the Tukey's test.

No differences were found between the exposure of leaf disks to 500 or 1,000 μM of glyphosate. At 1000 μM, the NT population of *C. elata* presented 4.6-fold more shikimic than the T population. However, the T populations of *C. barbata* and *C. ciliata* showed no differences, accumulating only 1.13 and 1.07-fold more shikimic acid, respectively, than their respective NT populations (Figure 3).

It was determined that *C. barbata* and *C. ciliata* exhibited natural tolerance to glyphosate, and the T and NT populations of *C. elata* were renamed resistant (R) and susceptible (S) to glyphosate, respectively. In the following assays, we focused in *C. elata*.

### Absorption and translocation

**Figure 4:**
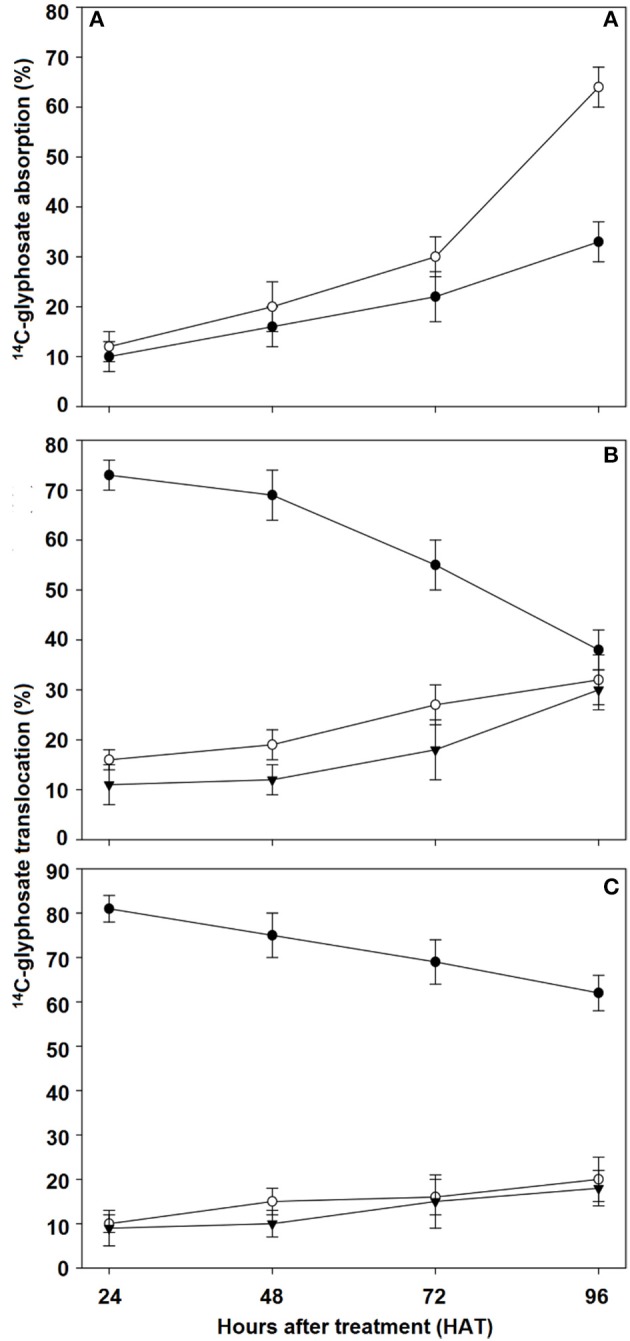
^14^C-glyphosate absorption and translocation in susceptible (S) and resistant (R) *Chloris elata* populations from 24 to 96 h after glyphosate treatment. **(A)** ^14^C-glyphosate absorption in S (○) and R (•) plants. ^14^C-glyphosate translocation in S **(B)** and R **(C)** plants. Treated leaf (•), remaining shoot tissue (○), and roots (▾). Vertical bars are ± standard errors (*n* = 5).

Total ^14^C-glyphosate recovery was 93.2 and 94.3% for the R and S populations of *C. elata*, respectively (data not shown). ^14^C-glyphosate absorption increased slowly in the first 72 HAT. At this time, population S had absorbed 30% of glyphosate, while population R had only absorbed 22%. The maximum glyphosate absorption rate was observed between 72 and 96 HAT, which was the two-fold higher in the S population (64%) than in the R population (33%; Figure 4A).

In both populations, ^14^C-glyphosate levels in treated leaves of *C. eleta* declined from 24 to 96 HAT, with the rate of movement out of the treated leaf greater and faster in the S population than in R population. The initial amount quantified of 73% at 24 HAT in the treated leaf decreased to 38% at 96 HAT in S population. Conversely, the ^14^C-glyphosate was retained mainly in the leaf treated in the R population, dropping from 81 to 62% at 24 and 96 HAT, respectively. An average of 32 and 30% of the glyphosate translocated reached the remaining shoot tissue and roots at 96 HAT in the S population, whereas in R population it was only of 20 and 18%, respectively (Figures 4B,C).

**Figure 5:**
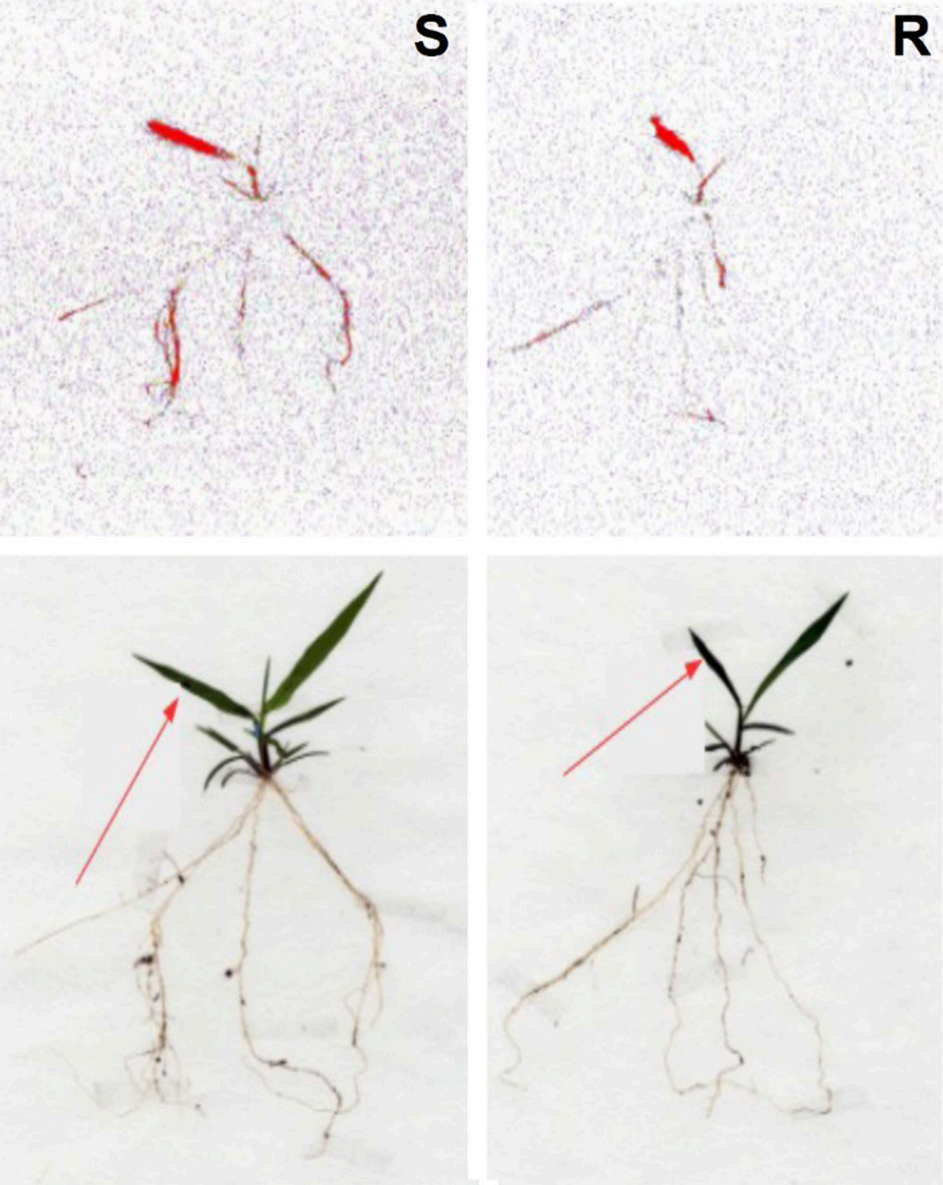
Translocation of ^14^C-glyphosate in susceptible **(S)** and resistant **(R)** *Chloris elata* plants at 96 h after application. The highest concentration of ^14^C-glyphosate is highlighted in red. Arrows indicate the treated leaf.

The ^14^C-glyphosate visualization by phosphor imaging revealed differences in the distribution between the S and R populations of *C. elata*. There was a difference in the translocation of glyphosate from treated leaves to shoots and roots, and the S population translocated higher amounts of ^14^ C-glyphosate compared to the R population (Figure 5).

### Glyphosate metabolism

For glyphosate absorbed into the R and S *C. elata* populations, much of the herbicide was unaltered in plants by 96 HAT. At this time, 89.4 and 91.0% of the applied herbicide remained as glyphosate in plants of the R and S populations, respectively. The levels of AMPA were 7.2 and 6.4%, while glyoxylate levels reached 3.4 and 2.6% in the R and S plants, respectively. For both AMPA and glyoxylate, these differences between the R and S populations were non-significant (*P* = 0.8741 for AMPA, and *P* = 0.6318 for glyoxylate).

### EPSPS enzyme activity assays

**Figure 6:**
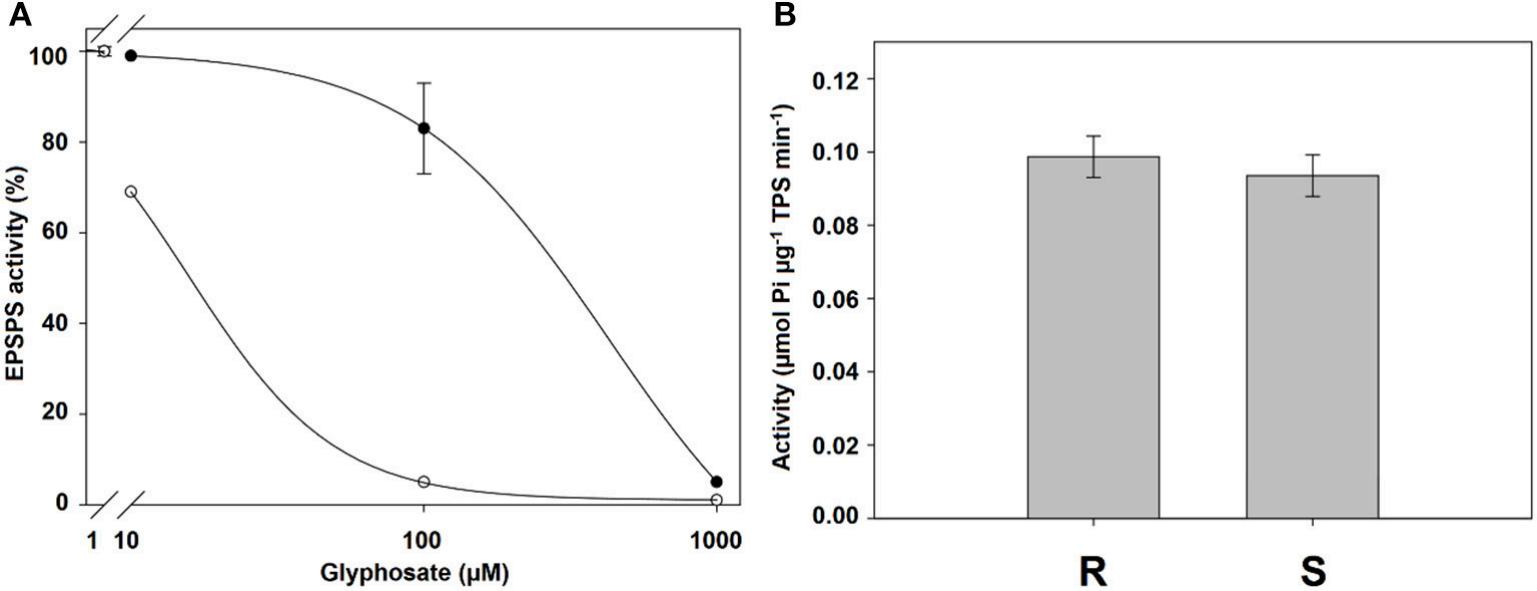
**(A)** EPSPS enzyme activity expressed as percentage of the untreated control in leaf extracts of plants from glyphosate-susceptible (○) and -resistant (•) *Chloris elata* plants. **(B)** Basal EPSPS activity for *C. elata* populations. Vertical bars are ± standard errors (*n* = 3).

The R population was 27.3-fold more resistant than the S population. The basal enzyme activity showed no differences between populations with 0.0987 to 0.0937 mmol mg^−1^ TPS^−1^ min^−1^ for the R and S populations, respectively (Figure 6).

### Sequencing of the EPSPS gene

**Figure 7:**
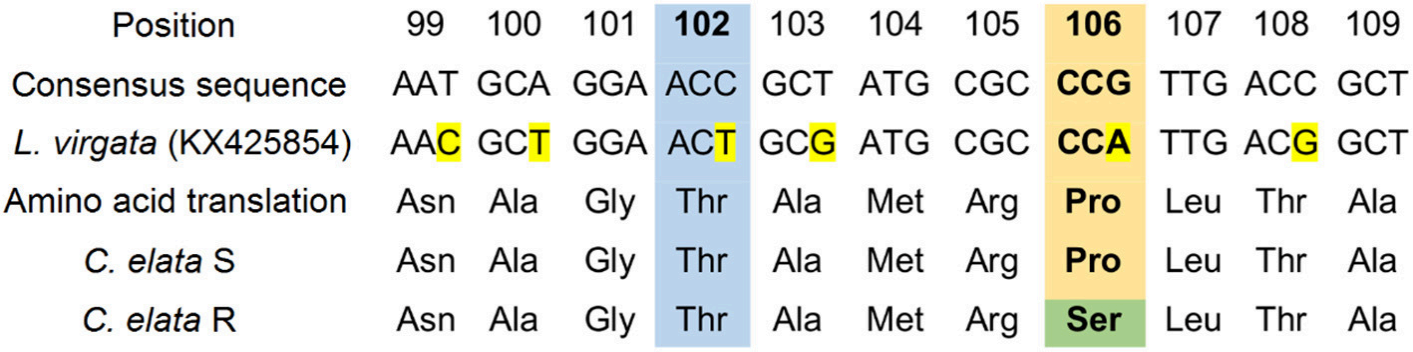
Partial alignment of nucleotides and amino acid sequences of EPSPS genes between glyphosate-susceptible and -resistant *Chloris elata* populations and *Leptochloa virgata* (GenBank: KX425854). The yellow color indicates nucleotide changes among species. The green color indicates an amino acid substitution at 106 position from Proline (CCG) to Serine (TCG). Box includes the 102 (blue) and 106 (orange) positions (amino acid number based on the start codon (ATG) of *Arabidopsis thaliana*, corresponding to point mutations associated for conferring glyphosate resistance.

A total of 120 bp of the EPSPS gene of the R and S *C. elata* populations was sequenced. The fragments were aligned and numbered based on a published EPSPS sequence of *Leptochloa virgata* (GenBank: KX425854) (Alcántara-de la Cruz et al., 2016c). Protein alignment of the predicted EPSPS fragments from R and S populations of *C. elata* showed 91.6 and 92.5% protein similarity, respectively, to that of *L. virgata*. The R population of *C. elata* showed an amino acid substitution at position 106 consisting of a proline to serine change. The substitution resulted in a TCG (serine) codon instead of a CCG (proline) codon (Figure 7).

## Discussion

The three *Chloris* populations from citrus orchards in Cuba were identified as *C. barbata, C. ciliata*, and *C. elata*. The number of species of *Chloris* recognized by different authors in Mexico and in the nearby Caribbean Islands is variable (Barkworth, 2007; Cerros-Tlatilpa et al., 2015). In these regions, the most frequent species are *C. ciliata, C. elata, C. barbata*, and *C. virgata* (Cerros-Tlatilpa et al., 2015). In Cuba, 12 species of the *Chloris* genus have been found (Catasús-Guerra, 2002).

The AFLP-based classification of *Chloris* populations revealed molecular-based relationships between three basic entities, which closely matched the morphology-based identification of three different species. The selected AFLP markers can be useful candidates in the pursuit of disentangling phylogenetic relationships among *Chloris* species. However, although these markers allowed to separate the T and NT populations of *C. elata*, they cannot be used to detect glyphosate resistance, because could produce biased results due to fragment-size homoplasy (Caballero et al., 2008).

To characterize the glyphosate susceptibility in *Chloris* species, it is important to consider the innate tolerance to this herbicide that has been observed in some species of the genus. Depending on the species studied, the LD_50_ (% survival plant) values can vary between 515 and 703 g ae ha^−1^ (Ngo et al., 2017a,b). These values are lower than those we have found for *C. barbata* and *C. ciliata*, including the populations never exposed to this herbicide, demonstrating an innate tolerance to glyphosate in these two species. Similar results were described for *Chloris polydactyla* from Brazil, where even some accessions with no history of applications presented lower susceptibility to glyphosate than those accessions with a history of glyphosate applications (Barroso et al., 2014). Innate tolerance has been well studied in grass weeds (Fernández-Moreno et al., 2016), and leguminous species (Cruz-Hipolito et al., 2009, 2011; Rojano-Delgado et al., 2012; Alcántara-de la Cruz et al., 2016a; Mao et al., 2016). The mechanism proposed is a lack of ^14^C-glyphosate absorption and/or translocation in tolerant plants compared to susceptible ones.

*C. elata* has a different profile than *C. barbata* and *C. ciliata*, and its GR_50_ and LD_50_ values demonstrate a clear quantitative difference between those plants harvested from T fields compared to those plants from NT fields. The lower shikimic accumulation by the T *C. elata* population (4.9 times) compared to the NT population showed the greatest resistance level of this species, similar to other *Chloris* species, such as *C. elata* (5.4) from Brazil (Brunharo et al., 2016) and *C. virgata* (2.0–9.7) and *Chloris truncata* (2.4–8.7) from Australia (Ngo et al., 2017a,b). When glyphosate is applied via foliar application, the EPSPS enzyme is inhibited, and there is a rapid accumulation of shikimate (Shaner et al., 2005). The amount of glyphosate in the NT population of *C. elata* determined the inhibition of EPSPS and rapid shikimate accumulation demonstrating the high susceptibility (S) of this population. Therefore, the T population of *C. elata* was characterized as resistant (R) to glyphosate, and the populations T and NT of *C. barbata* and *C. ciliata* are tolerant to this herbicide. These results are reflected in those obtained in dose response assays. For this reason, we continued to study the glyphosate resistance mechanisms only in the case of *C. elata*.

To date, few cases of reduced glyphosate absorption and/or translocation have been studied as a mechanism of resistance in the genus *Chloris*, and the results are contradictory. ^14^C-glyphosate studies on *C. virgata* and *C. truncata* do not show significant differences in the absorption and subsequent translocation of the herbicide, and the resistance was determined by mechanisms within the target site (Ngo et al., 2017a,b). However, another study on *C. elata* shows that lower glyphosate absorption and translocation in the R population are the only mechanisms involved in its glyphosate resistance (Brunharo et al., 2016). In our case, the R *C. elata* population collected in Cuba shows a resistance mechanism similar to that previously found for the species. Thus, ^14^C-absorption and translocation are higher in the S population than in the R population. These results suggest that less absorption and translocation contributed to the resistance to glyphosate in R *C. elata* plants.

Glyphosate metabolism has thus far not been identified as a major mechanism of resistance in plants, but is likely the result of plants not succumbing to glyphosate because of the expression of another resistance mechanism (Sammons and Gaines, 2014; Fernández-Moreno et al., 2017a). Only in a few cases has metabolism been demonstrated to be a secondary mechanism in glyphosate resistance, because in these cases, other major mechanisms are involved (Bracamonte et al., 2016). Our research substantiates that of other studies, with <90% of the absorbed glyphosate remaining unaltered in R and S plants of *C. elata*. It is likely that the ability of grass weeds to metabolize glyphosate is diminished once EPSPS is inhibited (González-Torralva et al., 2012; Fernandez et al., 2015). Considering the small extent of glyphosate metabolism, the significance of this result is unlikely biologically meaningful in the resistance to glyphosate in *C. elata*.

The I_50_ values were significantly different between the *C. elata* populations. The R population exhibited a high resistance level compared to the S population. The results with high I_50_ values and low shikimic acid values, as has already been explained and demonstrated in other studies, are associated with alterations in the gene encoding the herbicide target protein (Sammons and Gaines, 2014; Yu et al., 2015). Then, the TSR mechanism could be involved in the resistance to this species. Similar results have been shown in other weed species, including *L. virgata* (Alcántara-de la Cruz et al., 2016c), *Lolium multiflorum* (Salas et al., 2015), and *L. rigidum* (Fernandez et al., 2015). In these cases, higher I_50_ values, as well as the higher basal activity of EPSPS, were found in the resistant populations compared to the susceptible populations. It was thought that an overexpression of EPSPS played a role as a resistance mechanism (Ngo et al., 2017a). However, there were no significant differences in the basal activity of EPSPS between R and S populations of *C. elata*, precluding the involvement of such a mechanism.

The EPSPS sequence alignment showed only a mutation point at position Pro-106-Ser in the R *C. elata* population. Four substitutions in this genomic EPSPS position (Pro-106-Ser, Pro-106-Thr, Pro-106-Ala, and Pro-106-Leu), have been reported in mono- and dicotyledonous weeds, endowing resistance to glyphosate (Sammons and Gaines, 2014). A mutation to a different amino acid at this point causes a structural change in the target site, shifting the other amino acids toward the inhibitor by reducing the available space (Healy-Fried et al., 2007). These explain the resistance of the R population of *C. elata* at a molecular level. Some grassweed species which have shown a mutation at Pro-106 position are: *C. virgata* (Ngo et al., 2017a), *Echonoclhoa colona* (Alarcón-Reverte et al., 2015), *L. virgata* (Alcántara-de la Cruz et al., 2016c), *L. rigidum* (Fernandez et al., 2015), and *Poa annua* (Cross et al., 2015), among others.

## Conclusions

Morphological- and molecular-based analysis allowed the identification of the three *Chloris* species collected in citrus orchards from central Cuba. *C. barbata* and *C. ciliata* were characterized as being innately tolerant to glyphosate, and *C. elata* was identified as resistant to this herbicide. The last species had non-target site (reduced absorption and translocation) and target site (Pro-106-Ser mutation) resistance mechanisms to glyphosate.

These results confirm the first case of herbicide resistance in Cuba and strongly suggest that species of the *Chloris* genus can be either resistant or tolerant to glyphosate, supporting the previous reports of both glyphosate statuses in this genus.

## Author contributions

EB and RDP: Idea and designed the experiments; EB, PF-M, MO, FB, HC-H, and RA-dlC: Performed the research. PF-M, FB, MO, RA-dlC, and RDP; Analyzed the results. All authors contributed to write and approve the manuscript.

### Conflict of interest statement

The authors declare that the research was conducted in the absence of any commercial or financial relationships that could be construed as a potential conflict of interest.
